# Engineering of a Spider Peptide *via* Conserved Structure-Function Traits Optimizes Sodium Channel Inhibition *In Vitro* and Anti-Nociception *In Vivo*

**DOI:** 10.3389/fmolb.2021.742457

**Published:** 2021-09-21

**Authors:** H. Hu, S. E. Mawlawi, T. Zhao, J. R. Deuis, S. Jami, I. Vetter, R. J. Lewis, F. C. Cardoso

**Affiliations:** ^1^ Institute for Molecular Bioscience, The University of Queensland, Brisbane, QLD, Australia; ^2^ School of Pharmacy, The University of Queensland, Brisbane, QLD, Australia; ^3^ Centre for Innovations in Peptide and Protein Science, The University of Queensland, Brisbane, QLD, Australia

**Keywords:** spider peptide, sodium channel, peptide engineering, rational design, optimization, neurological diseases, chronic pain, therapy

## Abstract

Venom peptides are potent and selective modulators of voltage-gated ion channels that regulate neuronal function both in health and in disease. We previously identified the spider venom peptide Tap1a from the Venezuelan tarantula *Theraphosa apophysis* that targeted multiple voltage-gated sodium and calcium channels in visceral pain pathways and inhibited visceral mechano-sensing neurons contributing to irritable bowel syndrome. In this work, alanine scanning and domain activity analysis revealed Tap1a inhibited sodium channels by binding with nanomolar affinity to the voltage-sensor domain II utilising conserved structure-function features characteristic of spider peptides belonging to family NaSpTx1. In order to speed up the development of optimized Na_V_-targeting peptides with greater inhibitory potency and enhanced *in vivo* activity, we tested the hypothesis that incorporating residues identified from other optimized NaSpTx1 peptides into Tap1a could also optimize its potency for Na_V_s. Applying this approach, we designed the peptides Tap1a-OPT1 and Tap1a-OPT2 exhibiting significant increased potency for Na_V_1.1, Na_V_1.2, Na_V_1.3, Na_V_1.6 and Na_V_1.7 involved in several neurological disorders including acute and chronic pain, motor neuron disease and epilepsy. Tap1a-OPT1 showed increased potency for the off-target Na_V_1.4, while this off-target activity was absent in Tap1a-OPT2. This enhanced potency arose through a slowed off-rate mechanism. Optimized inhibition of Na_V_ channels observed *in vitro* translated *in vivo*, with reversal of nocifensive behaviours in a murine model of Na_V_-mediated pain also enhanced by Tap1a-OPT. Molecular docking studies suggested that improved interactions within loops 3 and 4, and C-terminal of Tap1a-OPT and the Na_V_ channel voltage-sensor domain II were the main drivers of potency optimization. Overall, the rationally designed peptide Tap1a-OPT displayed new and refined structure-function features which are likely the major contributors to its enhanced bioactive properties observed *in vivo*. This work contributes to the rapid engineering and optimization of potent spider peptides multi-targeting Na_V_ channels, and the research into novel drugs to treat neurological diseases.

## Introduction

Animal venoms are an exquisite source of bio-active peptides that modulate human neurophysiology. Spider venoms, in particular, are rich in inhibitory cysteine knot (ICK) peptides that often modulate voltage-gated ion channels in pain pathways (Cardoso et al., 2018; Cardoso and Lewis, 2018). These ICK peptides bind to the voltage-sensor domains of ion channels and modify their gating properties to open and close the ion-selective pore at non-conventional membrane potentials, and are hence named gating modifiers (Cardoso and Lewis, 2019). Amongst known spider gating-modifiers are potent modulators of voltage-gated sodium (Na_V_) channels such as the ProTx family, Df1a, Tap1a, Hm1a and Pn3a (Priest et al., 2007; Schmalhofer et al., 2008; Cardoso et al., 2015; Cardoso et al., 2017; Deuis et al., 2017; Cardoso et al., 2021), with a few simultaneously modulating the low-voltage T-type calcium (Ca_V_3) channels (Bladen et al., 2014; Cardoso et al., 2017; Cardoso et al., 2021) and the voltage-gated potassium (K_V_) channels (Middleton et al., 2002).

The Na_V_ channel family comprises nine subtypes (Na_V_1.1–Na_V_1.9) composed of an α-subunit associated with one or more auxiliary subunits β1 to β4 (Cardoso and Lewis, 2018). These channels are widely expressed in the central and peripheral nervous system, with subtypes Na_V_1.4 and Na_V_1.5 expressed in skeletal and cardiac muscles. Alterations in Na_V_ channel function contribute to a range of neurological disorders including chronic pain, epilepsy, and motor neuron disease (Kubat Oktem et al., 2016; Catterall, 2017; Cardoso and Lewis, 2018; Saba et al., 2019; Cardoso, 2020). These alterations are presented as remodelling of the expression and excitability of the subtypes Na_V_1.1, Na_V_1.3, Na_V_1.6, Na_V_1.7, Na_V_1.8 and Na_V_1.9 (Cardoso and Lewis, 2018; Cardoso, 2020). Chronic visceral pain is a highly prevalent problem associated to irritable bowel syndrome (IBS) that affects 11% of the global population (Chey et al., 2015; Enck et al., 2016), and in which Na_V_ channel subtypes were shown to participate *via* signalling visceral pain in IBS (Feng et al., 2015; Osteen et al., 2016; Erickson et al., 2018; Grundy et al., 2018; Salvatierra et al., 2018). Spider peptides targeting Na_V_ channels have shown promising pre-clinical therapeutic effects in reverting some of these Na_V_-related complex conditions. For example, the peptides ProTx-II, HwTx-IV and Tap1a showed analgesic effects in painful diabetic neuropathy, spared nerve injury-induced neuropathy and chronic visceral pain (Liu et al., 2014; Tanaka et al., 2015; Flinspach et al., 2017; Cardoso et al., 2021), respectively, and the peptide Hm1a reduced seizures in Dravet syndrome (Richards et al., 2018).

Studies of the structure-function relationships of Na_V_-targeting spider peptides have guided the design of optimized leads with improved potency and selectivity for Na_V_ channel subtypes (Cardoso and Lewis, 2019). Key residues involved in channel inhibition and selectivity and conserved amongst spider peptides of the same NaSpTx family have been unravelled (Klint et al., 2012; Cardoso and Lewis, 2019). For example, NaSpTx1 peptides often bind to the voltage-sensor domain II (VSDII) of Na_V_ channels to engender ion channel inhibition, as observed for HwTx-IV (Xiao et al., 2008), CcoTx-1 (Shcherbatko et al., 2016), Df1a (Cardoso et al., 2017) and Hd1a (Klint et al., 2015). Studies using residue substitutions demonstrated the positions 5 or 6 in the loop 1 of these ICK structures are typically occupied by phenylalanine and are critical for Na_V_ inhibition (Cardoso and Lewis, 2019). In addition, studies of activity optimization in NaSpTx1 peptides introduced a decrease in negative charge at the N-terminus (Minassian et al., 2013; Revell et al., 2013; Rong et al., 2013; Shcherbatko et al., 2016; Rahnama et al., 2017; Mueller et al., 2020), with such introduction also described to increase the ability of these peptides to bind to the cell membrane (Henriques et al., 2016; Mueller et al., 2020). At the C-terminal, residue modifications that enhanced Na_V_ inhibition introduced positively charged and/or hydrophobic residues (Minassian et al., 2013; Revell et al., 2013; Shcherbatko et al., 2016). Although these optimized leads have shown enhanced *in vitro* pharmacology, the translation into improved benefits *in vivo* in pre-clinical models of pain remain to be achieved for these peptide leads (Cardoso et al., 2017; Agwa et al., 2020).

In this work, we investigated the structure-function relationships of the spider peptide Tap1a by using pharmacological studies with the VSDII of Na_V_1.1 and by evaluating the impact of alanine residue substitutions on the activity of Tap1a at Na_V_1.1 and Na_V_1.7. We designed two optimized versions of Tap1a based on the enhanced structure-function properties of other NaSpTx1 peptides and characterized their activities at Na_V_ channels and their binding mode to VSDII. Finally, we investigated if the optimization of Na_V_ inhibition observed *in vitro* translated *in vivo* in the reversal of nocifensive behaviours in a murine model of Na_V_-induced spontaneous pain. This work contributes to the understanding of the structure-function relationships and optimization of spider peptides targeting Na_V_ channels, and the development of useful therapeutic leads with promising enhanced *in vivo* benefits for the treatment of neurological disorders.

## Results

### Tap1a Binds to VDSII S3–S4 Loop of Na_V_1.1

**FIGURE 1:**
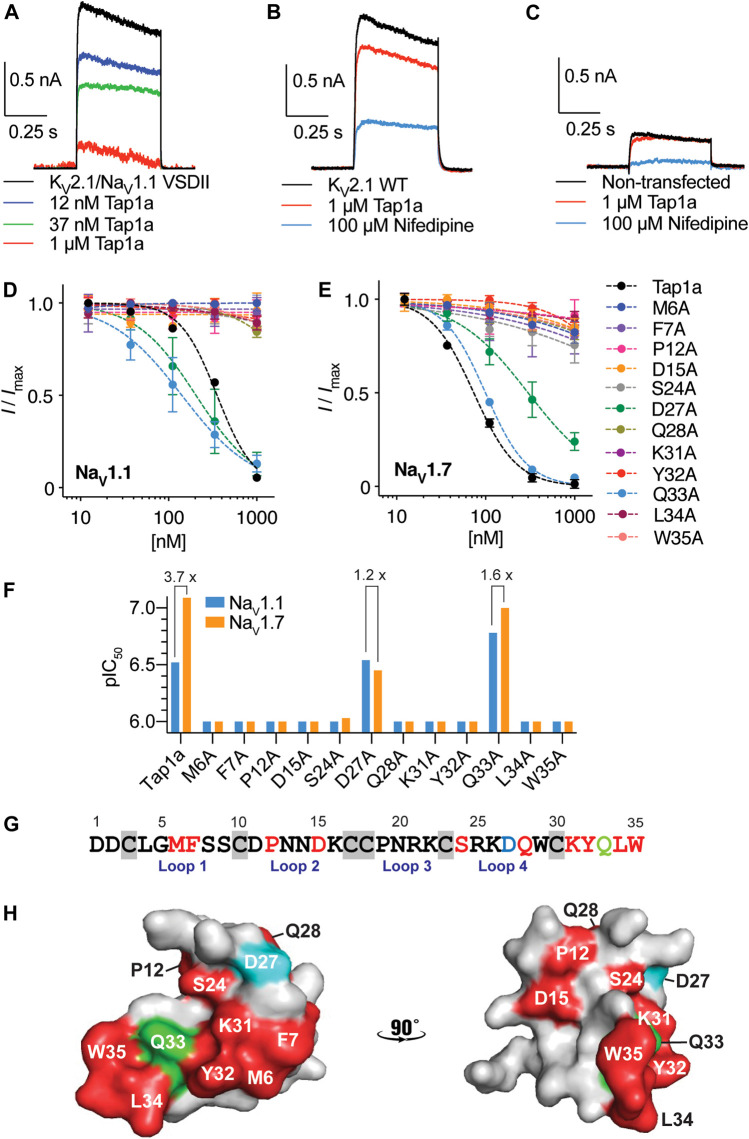
Binding site of Tap1a at hNa_V_1.1 and pharmacological properties of Tap1a alanine mutants for hNa_V_1.1 and hNa_V_1.7 channels. **(A–C)** A rK_V_2.1/hNa_V_1.1 chimera containing the paddles S3–S4 from DII from Na_V_1.1 was used to explore the binding site of Tap1a over Na_V_1.1. Potassium currents were elicited by a pulse at +20 mV for 500 ms from –90 mV holding potential. **(A)** The K^+^ currents are shown before and after addition of increasing concentrations of Tap1a. **(B)** Tap1a had weak effect on wild type rK_V_2.1 at 1 μM. **(C)** Tap1a had no effect on endogenous K^+^ currents from non-transfected HEK293 cells. Data are representatives from *n* = 3–5 independent experiments for each condition assayed, one whole cell was considered per independent experiment. **(D, E)** Tap1a and alanine mutants produced by recombinant expression were characterized *via* automated electrophysiology using QPatch 16X and HEK293 cells expressing hNa_V_1.1 **(D)** or hNa_V_1.7 **(E)** co-expressed with the β1 subunit. Sodium currents were elicited by depolarization to 0 mV for 20 ms from a pre-pulse at –120 mV for 200 ms and –80 mV holding potential. Serial diluted concentrations of each alanine mutant were incubated with the cells for 5 min at holding potential before channel activation. Data are described by mean ± SEM from *n* = 3–5 experiments. The calculated IC_50_ values are described in Table 1 **(F)** pIC_50_ values for Tap1a and alanine mutants tested against hNa_V_1.1 and hNa_V_1.7 and calculated from the dose responses produced in **(D)** and **(E)**. The selectivity between hNa_V_1.1 and hNa_V_1.7 are described above the bars when applicable. **(G)** Primary sequence of Tap1a highlighting in red residues which alanine substitution deplete the inhibitory activity, in blue the residue which alanine substitution led to a decrease in hNa_V_1.7 activity and maintained hNa_V_1.1 inhibitory activity, and in green the residue which alanine substitution led to an increase in hNa_V_1.1 activity and decrease in hNa_V_1.7 inhibitory activity. Cysteines are highlighted in grey boxes. **(H)** Model of the three-dimensional structure of Tap1a wild type highlighting in red the residues which were essential for hNa_V_1.1 and hNa_V_1.7 activity, in blue the residue D27 in which alanine substitution led to an increase or maintained inhibitory activity, and in green the residue Q33 in which alanine substitution led to a mixed increase and decrease in inhibitory activity.

Spider peptides belonging to NaSpTx1 are known to bind to VSDII of Na_V_ channels and induce gating-modifying effects that inhibit channel activation (Xiao et al., 2011; Cai et al., 2015). Tap1a displayed similar modulatory mechanism by inhibiting ion currents of the K_V_2.1/Na_V_1.1 VSDII chimera (Figure 1A) with a calculated IC_50_ value of approximately 185 nM (data not shown). Tap1a at 1 μM had weak effect on currents mediated by the wild type K_V_2.1 channel and reduced potassium currents by approximately 18% (area under the curve) (Figure 1B). These results suggested that the potent nanomolar inhibitory effects of Tap1a observed at the K_V_2.1/Na_V_1.1 VSDII chimera were due to its binding to the Na_V_1.1 VSDII S3-S4 loop. Non-transfected HEK293 cells had endogenous potassium currents measuring up to 0.3 nA that were not affected by Tap1a at up to 1 μM but were inhibited by 100 μM nifedipine (Figure 1C). Cells transfected with the chimera K_V_2.1/Na_V_1.1 VSDII produced potassium currents which were also sensitive to nifedipine at 100 μM (Supplementary Figure S1).

### Structure-Function Properties of Tap1a Resemble NaSpTx1 Peptides

In order to determine the structure-function relationships of individual residues in Tap1a, we applied the structure-function knowledge for NaSpTx1 (Cardoso and Lewis, 2019) and designed twelve alanine mutants of Tap1a: M6A, F7A, P12A, D15A, S24A, D27A, Q28A, K31A, Y32A, Q33A, L34A and W35A, and produced these new mutants *via* recombinant expression (Supplementary Table S1; Supplementary Figure S2). The recombinant expression of these peptides was achieved successfully and produced a major single isomer purified for further testing (Supplementary Figure S2). The molecular weight of the purified recombinant peptides was verified and confirmed by mass spectrometry (Supplementary Table S1; Supplementary Figure S2).

Tap1a alanine mutants were evaluated *via* automated whole cell patch-clamp electrophysiology with activities determined for the subtypes Na_V_1.1 and Na_V_1.7 (Figures 1D–G and Table 1). Residues identified as critical for Tap1a inhibitory activity were M6, F7, P12, D15, S24, Q28, K31, Y32, L34 and W35 in which alanine substitutions led to weaker or complete loss of activity at up to 1 μM peptide tested. The substitution D27A induced partial loss of activity at Na_V_1.7 (4.3-fold decrease) and did not affect activity at Na_V_1.1 compared to Tap1a-WT. Interestingly, the substitution Q33A increased the Tap1a inhibitory activity for Na_V_1.1 by 1.8-fold (not statistically significant) and had nearly no effect on the inhibition of Na_V_1.7 compared to Tap1a-WT (1.2-fold decrease).

**TABLE 1:** Pharmacological activity of Tap1a alanine mutants for hNa_V_1.1 and hNa_V_1.7 determined by automated electrophysiology in QPatch 16X. The values for the Tap1a wild type (WT) were obtained from our previous study (Cardoso et al., 2021). Tap1a alanine mutants were tested at up to 1 μM concentration.

Peptide	Na_V_1.1 IC_50_ (nM)			Na_V_1.7 IC_50_ (nM)		
	Mean	SEM	N	Mean	SEM	N
Tap1a-WT	301	42	7	80	6	4
M6A	>1000	n.a.	4	>1000	n.a.	3
F7A	>1000	n.a.	4	>1000	n.a.	3
P12A	>1000	n.a.	4	>1000	n.a.	3
D15A	>1000	n.a.	3	>1000	n.a.	3
S24A	>1000	n.a.	3	920	79	5
D27A	287	119	4	353	112	6
Q28A	>1000	n.a.	4	>1000	n.a.	4
K31A	>1000	n.a.	4	>1000	n.a.	3
Y32A	>1000	n.a.	3	>1000	n.a.	5
Q33A	163	54	5	99	4	5
L34A	>1000	n.a.	4	>1000	n.a.	3
W35A	>1000	n.a.	3	>1000	n.a.	8

The modulation of Na_V_1.1 and Na_V_1.7 by these alanine mutants affected the size of the Na^+^ peak current, and no alterations in the kinetics (*tau*) of fast inactivation were observed in the presence of these peptides under experimental conditions (data not shown). While Tap1a-WT displayed 3.7-fold preference for Na_V_1.7 over Na_V_1.1, the mutant D27A had 1.2-fold preference for Na_V_1.1 over Na_V_1.7, and Q33A had 1.6-fold preference for Na_V_1.7 over Na_V_1.1 (Figure 1F). The primary structure of Tap1a shows key residues for Na_V_-activity revealed from alanine scanning in the loops 1, 2 and 4 and the C-terminal regions (Figure 1G), while the three-dimensional model of Tap1a showed most of these residues clustered at one face of the peptide, while the residues P12, D15 and Q28 were located adjacent to this cluster (Figure 1H).

### Optimized Tap1a Displayed Increased Potency for Na_V_ Channels *In Vitro*

**FIGURE 2:**
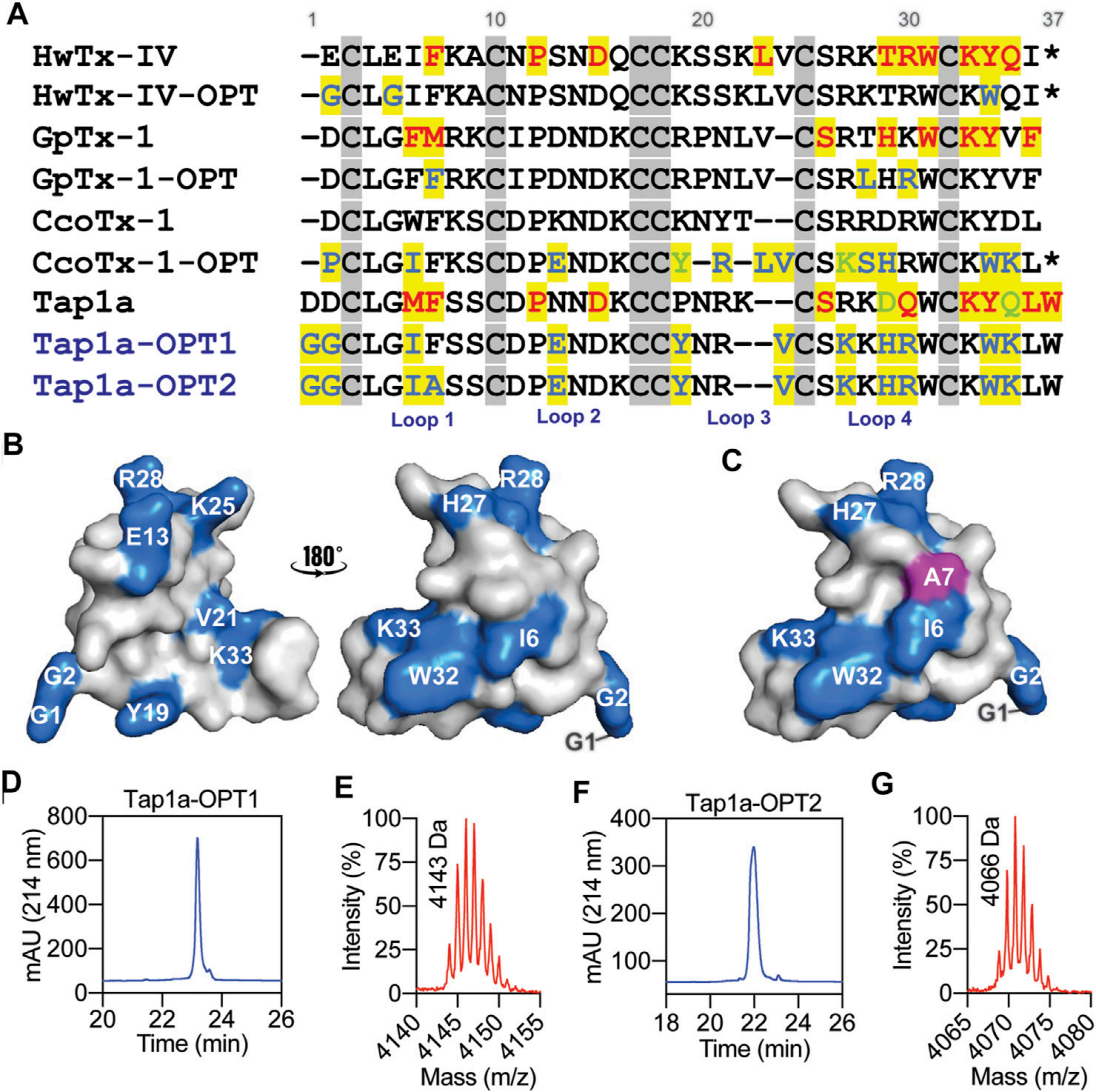
Rational design of Tap1a-OPT1 and Tap1a-OPT2, and peptide production *via* recombinant expression. **(A)** Alignment of NaSpTx1 spider peptides which were pharmacologically optimized for Na_V_ channels inhibition (Cardoso and Lewis, 2019), and Tap1a, Tap1a-OPT1 and Tap1a-OPT2. Residues coloured in red are positions where substitutions led to Na_V_-activity loss reported in previous studies and for Tap1a in this work, and residues coloured in blue and green are substitutions that enhanced potency and selectivity for Na_V_s, respectively. Residue substitutions in Tap1a-OPT1 and Tap1a-OPT2 are highlighted in blue. Cysteines are in grey boxes. **(B, C)** Model of the three-dimensional structures of Tap1a-OPT1 **(B)** and Tap1a-OPT2 **(C)** highlighting in blue the substitutions introduced in Tap1a-OPT1 and in pink the additional substitution F7A introduced in Tap1a-OPT2. **(D–G)** Production of the peptides Tap1a-OPT1 and Tap1a-OPT2. RP-HPLC chromatogram and mass spectrometry analysis results of the purified recombinants Tap1a-OPT1 **(D, E)** and Tap1a-OPT2 **(F, G)** purified in C18 column using a TFA/ACN gradient. Mass spectrometry analysis of purified peptides displayed the expected masses of 4143 Da for Tap1a-OPT1 **(E)** and 4066 Da for Tap1a-OPT2 **(G)**.

We designed and produced two optimized versions of the peptide Tap1a (Tap1a-WT) here named Tap1a-OPT1 and Tap1a-OPT2 (Figure 2). Our rational design focused on residue substitutions that warranted additional or new biochemical properties introduced to Tap1a-WT and were in line with the findings for the optimized peptides here named GpTx-1-OPT (Murray et al., 2015), HwTx-IV-OPT (Revell et al., 2013; Rahnama et al., 2017) and CcoTx-1-OPT (Shcherbatko et al., 2016). As a result of the rational design, Tap1a-OPT1 comprised the substitutions D1G, D2G, M6I, N13E, P19Y, K22V, R25K, D27H, Q28R, Y32W, Q33K, while Tap1a-OPT2 included the additional substitution F7A (Figures 2A–C). These newly designed peptides were successfully produced *via* recombinant expression and purified as described in Figures 2D–G.

**FIGURE 3:**
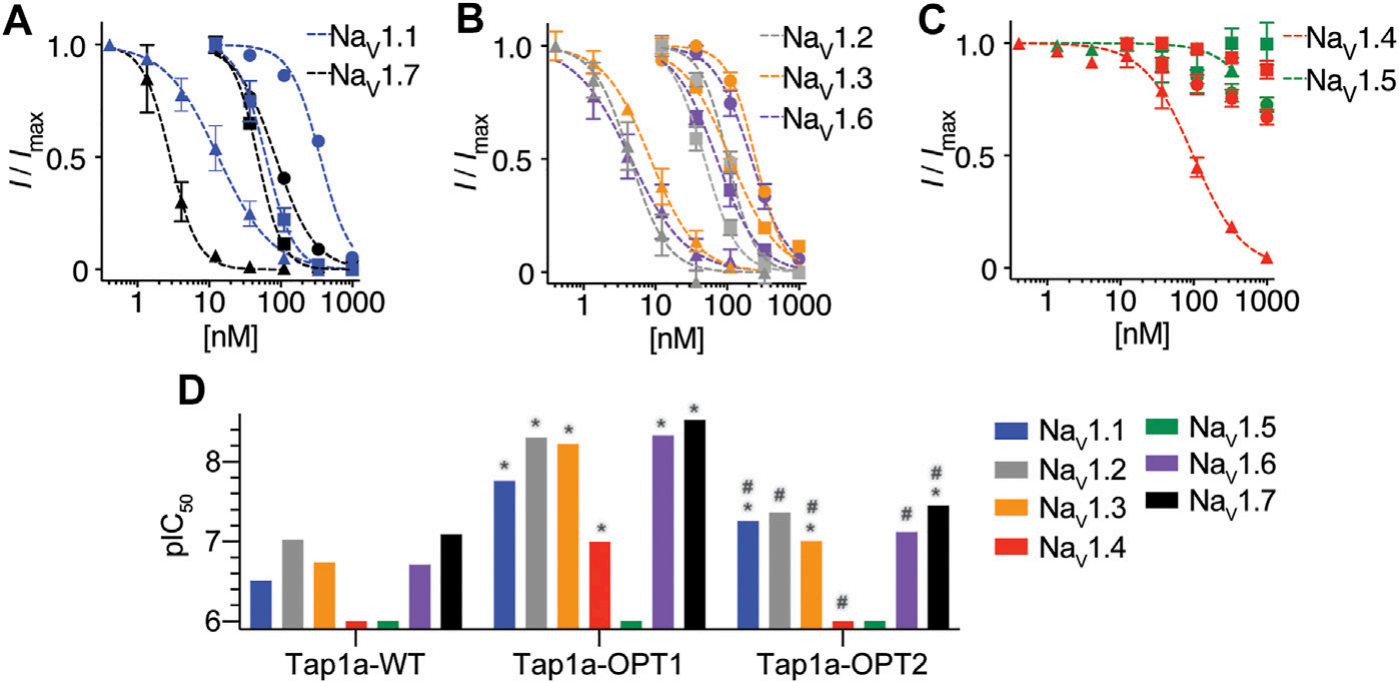
Na_V_ channel subtypes pharmacological profile of Tap1a-WT, Tap1a-OPT1 and Tap1a-OPT2 determined using automated electrophysiology. **(A–C)** Representative concentration-responses for the inhibition of the hNa_V_1.1 to hNa_V_1.7 channels in the presence of increasing concentrations of Tap1a-WT (circles), Tap1a-OPT1 (triangles) and Tap1a-OPT2 (squares) measured by automated whole-cell patch clamp in QPatch 16X. Holding potential was –80 mV and Na^+^ currents were elicited by 20 ms voltage step to 0 mV from a –120 mV conditioning pulse applied for 200 ms. The IC_50_ values calculated are described in Table 2 **(D)** Comparison of the pIC_50_ values for Tap1a-WT, Tap1a-OPT1 and Tap1a-OPT2 tested against hNa_V_1.1 to hNa_V_1.7 and calculated from the dose responses produced in **(A–C)**. *Statistically significant compared to Tap1a-WT; ^#^Statistically significant compared to Tap1a-OPT1; Individual *p* values are described in the text.

The ability of Tap1a-OPT1 and Tap1a-OPT2 to inhibit Na_V_ channels was measured *via* automated whole cell patch-clamp electrophysiology and their IC_50_ values determined for Na_V_1.1 to Na_V_1.7 channel subtypes (Figure 3; Table 2). Tap1a-OPT1 displayed a significant increase in potency for Na_V_1.1 and Na_V_1.7 (*p* = 0.0005 and *p* = 0.001, respectively), as well as for Na_V_1.2 (*p* = 0.01), Na_V_1.3 (*p* = 0.009) and Na_V_1.6 (*p* = 0.0003), and Na_V_1.4 (*p* < 0.00002) compared to Tap1a-WT, while no significant inhibition of Na_V_1.5 was observed at up to 1 μM (Figures 3A–D; Table 2). The substitution F7A in Tap1a-OPT1 produced Tap1a-OPT2 that displayed significant weaker Na_V_ inhibition compared to Tap1a-OPT1 for Na_V_1.1 (*p* = 0.02) and Na_V_1.7 (*p* = 0.0002), Na_V_1.2 (*p* = 0.0007), Na_V_1.3 (*p* = 0.002) and Na_V_1.6 (*p* = 0.007) (Figures 3A–D; Table 2), while Tap1a-OPT2 did not significantly inhibit Na_V_1.4 and Na_V_1.5 at up to 1 μM (Figure 3C; Table 2). Nonetheless, Tap1a-OPT2 maintained significant enhanced inhibition of Na_V_1.1 (*p* = 0.0009), Na_V_1.3 (*p* = 0.01), Na_V_1.6 (*p* = 0.004) and Na_V_1.7 (*p* = 0.004) compared to Tap1a-WT (Figures 3A–D; Table 2).

**TABLE 2:** Pharmacological activity of the spider peptide Tap1a-WT and optimized analogues Tap1a-OPT1 and Tap1a-OPT2 for hNa_V_ channels. The values for the Tap1a-WT were obtained from our previous study (Cardoso et al., 2021). Peptides were tested at up to 1 μM concentration.

Na_V_ subtype	Tap1a WT		Tap1a OPT1		Tap1a OPT2		Ratio WT/OPT1	Ratio WT/OPT2
	IC_50_ ± SEM (nM)	N	IC_50_ ± SEM (nM)	N	IC_50_ ± SEM (nM)	N		
Na_V_1.1	302 ± 43	7	17 ± 3*	5	54 ± 9* ^,^ ^#^	4	18	6
Na_V_1.2	95 ± 22	5	5 ± 1*	3	43 ± 5^#^	6	18	2
Na_V_1.3	180 ± 62	3	6 ± 0.5*	3	96 ± 9* ^,^ ^#^	4	27	2
Na_V_1.4	>1000	4	100 ± 19*	4	>1000^#^	4	>10	1
Na_V_1.5	>1000	3	>1000	3	>1000	4	1	1
Na_V_1.6	191 ± 28	8	4.6 ± 2.3*	4	75 ± 11* ^,^ ^#^	4	41	3
Na_V_1.7	81 ± 7	4	3 ± 0.7*	3	35 ± 2* ^,^ ^#^	4	27	2

Individual *p* values are described in the text.

^*^ Statistically significant compared to Tap1a-WT.

^#^ Statistically significant compared to Tap1a-OPT1.

Remarkable enhancement in inhibition by Tap1a-OPT1 was observed for the subtypes Na_V_1.1–Na_V_1.3, Na_V_1.6 and Na_V_1.7 with increases of 18 to 41-fold compared to Tap1a-WT (Table 2). Tap1a-OPT2 inhibited Na_V_ channels similarly to Tap1a-WT and displayed 2- to 6-fold potency increase compared to Tap1a-WT. Interestingly, Tap1a-OPT2 displayed a more selective pharmacology away from the off-targets hNa_V_1.4 and hNa_V_1.5 compared to Tap1a-WT as observed by the remaining Na^+^ currents in the presence of these peptides tested at up to 1 µM (Figure 3C).

### Enhanced Inhibitory Properties of Tap1a-OPT Occur *via* Decreased Off-Rate

**FIGURE 4:**
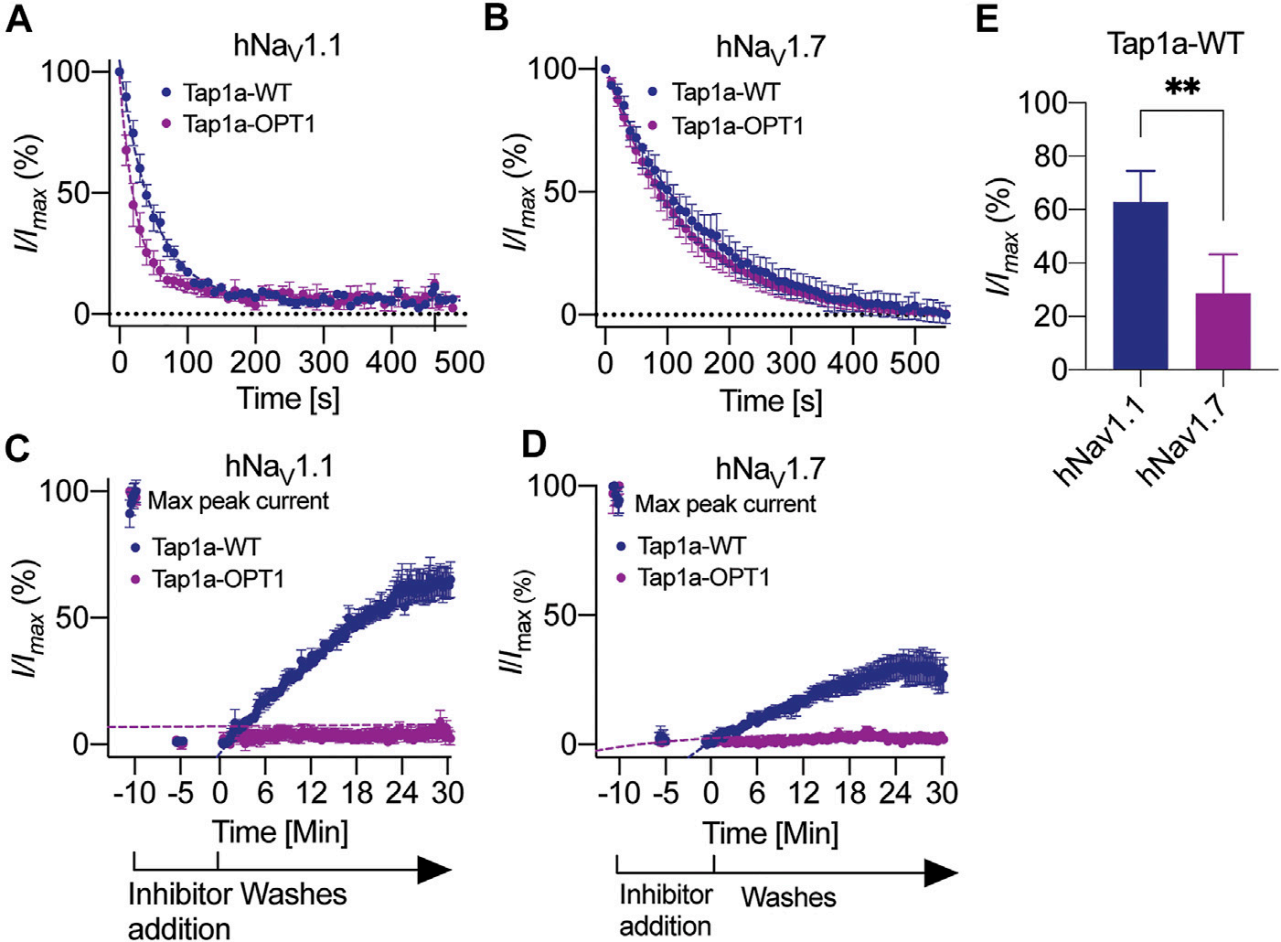
Kinetics of Na_V_1.1 and Na_V_1.7 inhibition by Tap1a-WT and Tap1a-OPT1. **(A, B)** On-rate measurements for Tap1a-WT and Tap1a-OPT1 over Na_V_1.1 **(A)** and Na_V_1.7 **(B)** were calculated from fitted exponentials of experiments recorded before and after addition of peptides at 10x IC_50_ concentration. **(C, D)** Off-rate measurements of Tap1a-WT and Tap1a-OPT1 over Na_V_1.1 and Na_V_1.7 were calculated from the fitted exponentials of experiments recorded before and after addition of peptides at 10x IC_50_ concentration followed by washes with extracellular solution. **(E)** Na_V_ currents measured at the end of 30 min for Na_V_1.l and 25 min for Na_V_1.7 ligand wash-out period. **p = 0.0042. Statistical significance was determined by *t*-student test. All data points are mean ± SEM of 4–7 independent experiments.

The rates of modulation of hNa_V_1.1 and hNa_V_1.7 by Tap1a-WT and Tap1a-OPT1 were measured in the presence of 10x the IC_50_ for each Na_V_ subtype (Figure 4). The on-rate evaluations revealed Tap1a-OPT1 produced a slight but significant increase in the on-rate for the hNa_V_1.1 subtype when compared to Tap1a-WT (Figure 4A and Table 3). Tap1a-OPT1 showed on-rate of 26 s at hNa_V_1.1 and was 1.9-fold faster than Tap1a-WT with on-rate of 48.4 s, and had a significant increase in K_*on*_ observed compared to Tap1a-WT. At hNa_V_1.7, Tap1a-OPT1 showed on-rate of 135 s and was 1.2-fold faster than Tap1a-WT with an on-rate of 163 s but not statistically significant (Figure 4B). Both Tap1a-WT and Tap1a-OPT1 produced statistically significant decreased K_on_ observed and faster on-rates to inhibit hNa_V_1.1 compared to hNa_V_1.7 (Figures 4A,B; Table 3). Tap1a-WT was 3.3-fold faster to inhibit hNa_V_1.1 compared to hNa_V_1.7, and Tap1a-OPT1 was 5.3-fold faster to inhibit hNa_V_1.1 compared to hNa_V_1.7.

**TABLE 3:** Kinetic properties of Tap1a-WT and Tap1a-OPT1 at hNa_V_1.1 and hNa_V_1.7 determined *via* patch-clamp electrophysiology. Kinetic data were determined using peptide concentrations equivalent to 10× IC_50_, and *k*_obs_ and *k*_off_ are given as the mean ± SEM from 4–7 independent experiments.

	K_on_ observed (s^−1^)	K_on_ (nM^−1^s^−1^)	K_off_ (s^−1^)	K_d_ (nM)
hNa_V_1.1				
Tap1a-WT	2.01 ± 0.1 × 10^−2^ ^†^	6.53 × 10^–6^	6.56 ± 0.32 × 10^−4^	3.16 × 10^–2^
Tap1a-OPT1	8.75 ± 0.8 × 10^−2^ * ^,^ ^#^	nd^b^	irreversible^a^	nd^b^
hNa_V_1.7				
Tap1a-WT	7.07 ± 1.2 × 10^−3^	6.52 × 10^–6^	8.46 ± 0.6 × 10^–4^	1.2 × 10^–1^
Tap1a-OPT1	8.60 ± 1.7 × 10^−3^	nd^b^	irreversible^a^	nd^b^

K_on_ observed = 1/τ_on_; k_on_ = [(1/τ_on_)–k_off_]/(ligand); k_off =_ 1/τ_off_; K_d_ = k_off_ /k_on_.

^*^ Statistically significant compared to Tap1a-WT K_on_ observed for Na_V_1.1, *p* = 0.0121.

^†^ Statistically significant compared to Tap1a-WT K_on_ observed for Na_V_1.7, *p* = 0.0061.

^#^ Statistically significant compared to Tap1a-OPT1 K_on_ observed for Na_V_1.7, *p* = 0.0012.

a K_off_ is less than the lowest valid measurement under the experimental conditions.

b nd = not determined.

The rates of dissociation of Tap1a-WT and Tap1a-OPT1 from hNa_V_1.1 and hNa_V_1.7 revealed Tap1a-OPT1 had a statistically significant decreased off-rate at both hNa_V_1.1 and hNa_V_1.7 subtypes compared to Tap1a-WT (Figures 4C,D; Table 3). While Tap1a-WT was washed off from hNa_V_1.7 with an off-rate of 19.7 min, Tap1a-OPT1 was nearly irreversible at the same experimental conditions and K_off_ was not able to be calculated. Similarly, the rates of dissociation from hNa_V_1.1 showed Tap1a-WT was washed off with an off-rate of 25.41 min, and Tap1a-OPT1 was nearly irreversible at the same experimental conditions. The irreversibility of hNa_V_1.1 inhibition by Tap1a-WT was more pronounced as per remaining Na^+^ currents measured at 30 min wash out and significantly higher than hNa_V_1.7 as described in Figure 4E.

### Tap1a-OPT1 Displayed Improved Target Engagement and Therapeutic Effects *In Vivo*

**FIGURE 5:**
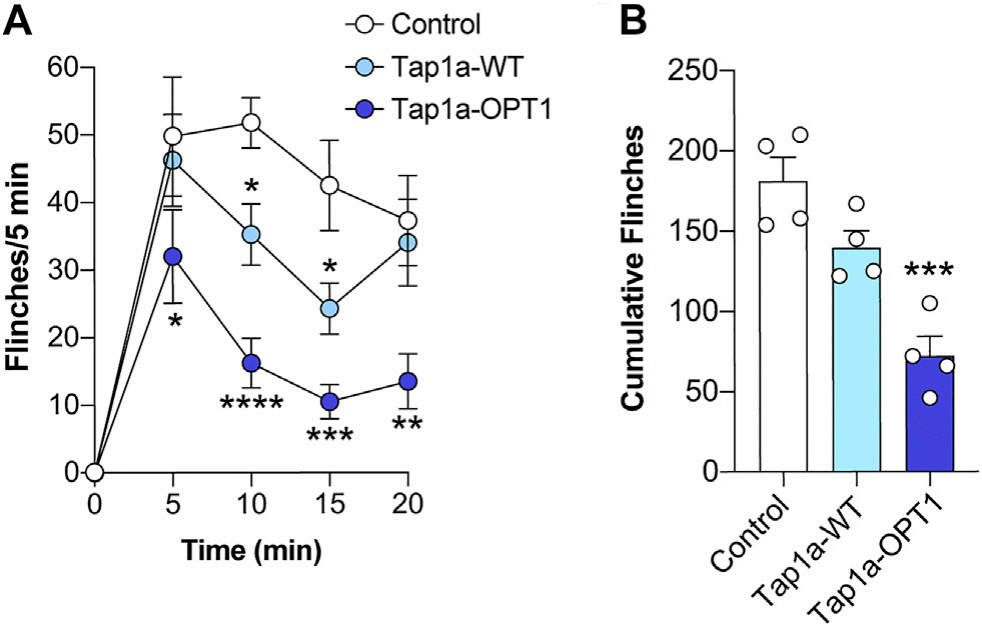
Tap1a-OPT1 has enhanced *in vivo* target engagement and antinociceptive effects. **(A)** Time course of spontaneous pain behaviors induced by intraplantar injection of the Na_V_1.7 activator OD1. Intraplantar injection of Tap1a wild-type (Tap1a-WT, 1 μM) or Tap1a-OPT1 (1 μM) significantly reduced OD1-induced spontaneous pain behaviors at the time points indicated. Statistical significance was determined by two-way ANOVA with Dunnett’s post-test. **(B)** Total OD1-induced pain behaviors over 20 min. Only Tap1a-OPT1 (1 μM) significantly reduced cumulative pain behaviors. Statistical significance was determined by one-way ANOVA with Dunnett’s post-test. Data is presented as mean ± SEM, *n* = 4 per mice per group. *p < 0.05, **p < 0.01, ***p < 0.001, ****p < 0.0001 compared to control.

To assess the efficacy of Tap1a-WT and Tap1a-OPT1 to inhibit Na_V_-induced nocifensive responses *in vivo*, we utilized a model of Na_V_1.7-mediated pain based on the intraplantar injection of the Na_V_1.7 activator OD1 (Deuis et al., 2016). Intraplantar injection of Tap1a-WT (WT, 1 μM) or Tap1a-OPT1 (OPT, 1 μM) significantly reduced OD1-induced spontaneous pain behaviors at the time points indicated (Figure 5A), albeit Tap1a-WT had a slower onset of action and was less effective compared to Tap1a-OPT1 (Figure 5B). In line with this, only Tap1a-OPT1 significantly reduced total pain behaviors, which is consistent with its improved potency at peripheral Na_V_s observed *in vitro* (cumulative flinches: control 181 ± 15; Tap1a WT 140 ± 10; Tap1a OPT1 72 ± 12; Figure 5B).

### Interactions of Tap1a-WT and Tap1a-OPT With Na_V_1.7 and Na_V_1.4

**FIGURE 6:**
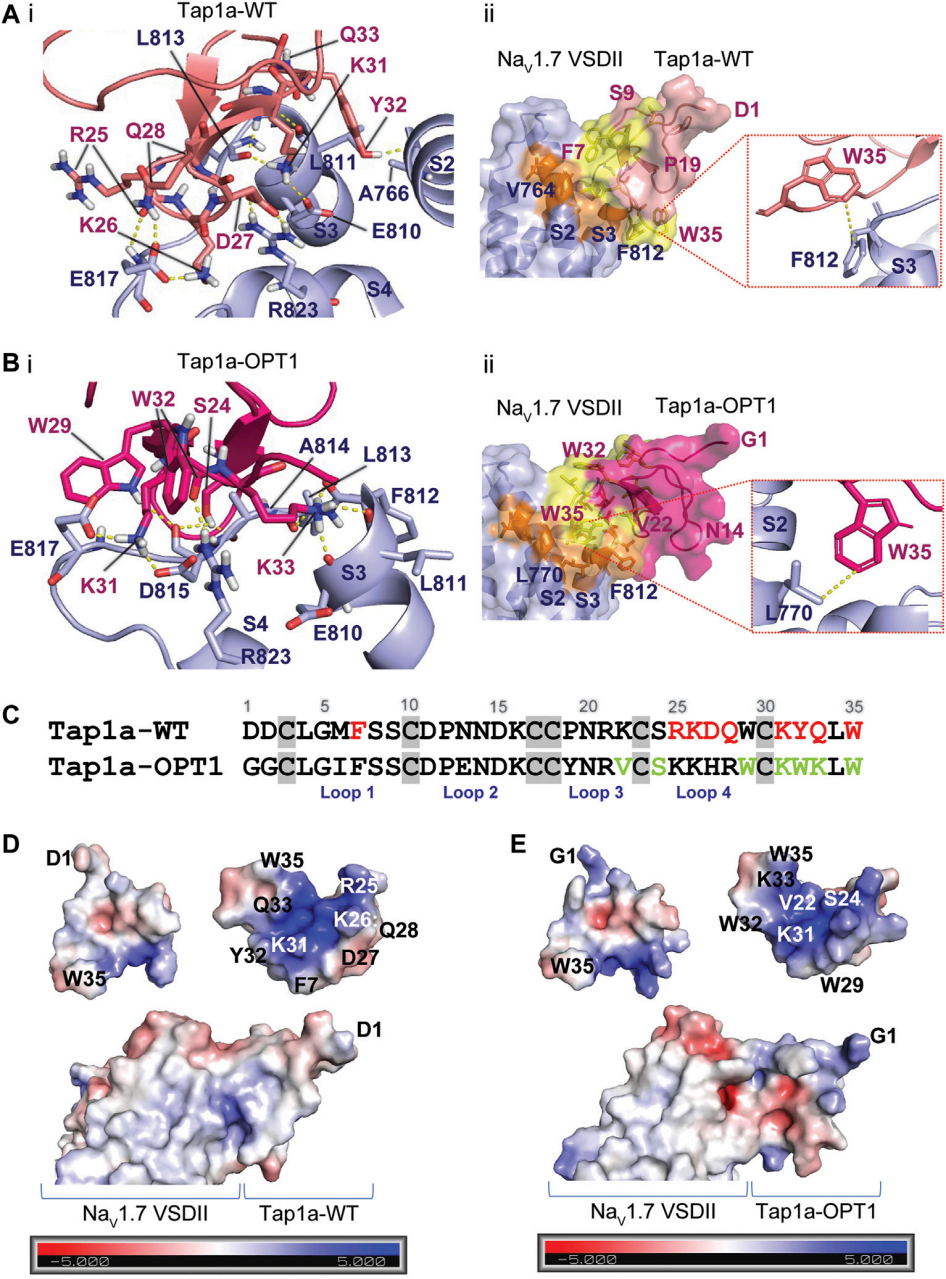
Molecular model of the Tap1a and Tap1a-OPT1 docked on the human Na_V_1.7 channel DII. **(Ai)** The three-dimensional representation of the interactive surface of Tap1a-WT (salmon) docked over the hNa_V_1.7 channel voltage sensor domain II / Na_V_Ab chimera (Na_V_1.7 DII) (light blue) are represented in cartoon. Side chains are represented in sticks. Hydrogen bonds with distances up to 3Å are represented by dashed lines colored in yellow. **(Aii)** Hydrophobic interacting surfaces of Tap1a-WT colored in yellow and the hNa_V_1.7 DII in orange. Zoom in at the Tap1a-WT C-terminal W35 showing a potential hydrophobic interaction with F812 in the S3 of Na_V_1.7 DII. Distance between the closet carbons of their aromatic rings is represented in yellow dashed lines. Side chains are represented by sticks. **(Bi)** The three-dimensional representation of the interactive surface of Tap1a-OPT1 (pink) docked over the hNa_V_1.7 DII (light blue) are represented in cartoon. Side chains are represented by sticks and hydrogen bonds with distances up to 3Å are represented by dashed lines colored in yellow. **(Bii)** Hydrophobic interacting surfaces of Tap1a-OPT1 colored in yellow and the hNa_V_1.7 DII in orange. Zoom in at the Tap1a-OPT1 C-terminal W35 showing a potential hydrophobic interaction with L770 in the S2 of Na_V_1.7 DII. Distance between the closet carbons of their side chains is represented in yellow dashed lines. Side chains are represented by sticks. **(C)** Primary structure of Tap1a-WT and Tap1a-OPT1 colored in red the residues predicted to form the strongest interactions between Tap1a-WT and hNa_V_1.7 DII, and in green the residues predicted to form the strongest interactions between Tap1a-OPT1 and hNa_V_1.7 DII. **(D, E)** Electrostatic surface of Tap1a-WT **(D)** and Tap1a-OPT1 **(E)**. Their predicted interactive ionic surface is visualized in the top right representations. The electrostatic potential of the interacting surfaces of Tap1a-WT or Tap1a-OPT1 with the Na_V_1.7 DII are visualized in the bottom representation of each figure. Electron density charge bars range from –5.0 (red) to +5.0 (blue).

The interactions of Tap1a-WT, Tap1a-OPT1 and Tap1a-OPT2 with Na_V_ channels were predicted by molecular docking using HADDOCK webserver (van Zundert et al., 2016) (Figure 6). Potential interactions between Tap1a-WT and Na_V_1.7 VSDII revealed strong hydrogen bonds (h-bonds) with distances ranging from (in Å) 1.5 to 2.9 (Figures 6Ai,C; Supplementary Table S2). Most h-bond interactions occurred between the loop 4 and the C-terminal of Tap1a-WT and the S2 and S3–S4 loop of Na_V_1.7 VSDII. Hydrophobic interactions with distances ranging from (in Å) 3.4 to 3.9 were observed between loop 1 and the C-terminal of Tap1a-WT and the S2 and S3–S4 loop of Na_V_1.7 VSDII (Figures 6Aii,C; Supplementary Table S2).

The predicted interactions of Tap1a-OPT1 with Na_V_1.7 VSDII revealed h-bonds with distances ranging from (in Å) 1.7 to 3.1 (Figures 6Bi,C; Supplementary Table S2). These occurred at loops 3 and 4 and C-terminal of Tap1a-OPT1 binding to the S3 and S3–S4 loop of the Na_V_1.7 VSDII. Hydrophobic interactions with distances ranging from (in Å) 3.2 to 4.5 were also observed between loops 3 and 4 and C-terminal of Tap1a-OPT1 and S2 and S3–S4 loop of Na_V_1.7 VSDII (Figures 6Bii,C; Supplementary Table S2). In an experiment that docked Tap1a-WT over Nav1.7 DII using the optimization restrains, we observed loops 3 and 4 and C-terminal forming the strongest h-bonds and hydrophobic interactions with the S3–S4 loop of the Na_V_1.7 VSDII (Supplementary Table S2).

The electrostatic surface of Tap1a-WT and Tap1a-OPT1 were calculated, as well as the electrostatic interacting surface of these peptides with the Na_V_1.7 VSDII as represented in Figures 6D,E. The interacting surface of Tap1a-OPT1 was favoured *via* residues that simultaneously form stronger hydrophobic or h-bonds interactions and additional electrostatic interactions with Na_V_1.7 VSDII, such as W32 and K33. Some of the Tap1a-WT predicted interacting residues forming hydrophobic or h-bonds are negatively charged or neutral, such as D27 and Q33, respectively, and likely produce opposed forces and weaker interactions with the negatively charged exposed surface of Na_V_1.7 VSDII.

In order to predict interactions with Na_V_1.4 VSDII S3–S4 loop that could explain the loss of Na_V_1.4 inhibition by Tap1a-OPT2, we docked Tap1a-OPT1 and Tap1a-OPT2 over Na_V_1.4 (Supplementary Table S2). Our results showed F7 in Tap1a-OPT1 or A7 in Tap1a-OPT2 likely do not interact with Na_V_1.4 VSDII. Instead, we observed an overall weakening of hydrophobic interactions between Tap1a-OPT2 and Na_V_1.4 VSDII (Supplementary Table S2). The docking between Tap1a-WT and Na_V_1.4 using the alanine scanning results as restrains also showed a potential reduction in hydrophobic interactions, while F7 was in the non-interacting surface of the Tap1a and away from the ion channel (Supplementary Table S2).

## Discussion

Venoms from species of spider, scorpion, cone snail and snake are unique cocktails of bioactive compounds with potential for novel drugs and therapies. These compounds range from small molecules to peptides and proteins, from which a few are approved and available for human use such as ziconotide derived from the ω-conopeptide MVIIA isolated from *Conus magus* and effective in the treatment of neuropathic pain (Deer et al., 2019), and captopril derived from proline-rich oligopeptides isolated from the Brazilian snake *Bothrops jararaca* and effective in the treatment of hypertension and congestive heart failure (Ferreira et al., 1970). While MVIIA was not required to undergo optimization to become ziconotide, the proline-rich oligopeptides derived from snake venom were optimized *via* structure-active relationship investigations that led to a small molecule derived from the terminal sulfhydryl moiety of these oligopeptides (Patlak, 2004). A number of venom-derived compounds have been investigated in pre-clinical trials (Pennington et al., 2018; Bordon et al., 2020) or are in more advanced stages of clinical trials such as chlorotoxin derivates for tumour painting in intraoperative visualization (Patil et al., 2019), and the ShK-186 analogue derived from the venom peptide ShK from sea anemone for the treatment of autoimmune diseases such as psoriatic arthritis and lupus (Pennington et al., 2015). These illustrate the exquisite potential of venom peptides and great feasibility of translation into novel therapeutics for human use.

### Tap1a has Conserved Structure-Function Properties as the NaSpTx1 Family

Recent advancements in the understanding of the structure-function relationships of Na_V_-targeting spider peptides allows a refined rational design of novel optimized inhibitors for the development of drugs and therapies (Cardoso and Lewis, 2019; Cardoso, 2020). Poorly treated diseases in which Na_V_ channels are proved to underly pathological processes, e.g., chronic pain, motor neuron disease and epilepsy, would benefit enormously from these novel optimized Na_V_-targeting molecules (Cardoso, 2020). The elucidation of the binding sites of spider-peptide inhibitors to Na_V_ channels revealed they preferably bind to the VSDII S3-S4 loop of these channels to engender inhibitory effects (Bosmans et al., 2008; Xiao et al., 2008; Shcherbatko et al., 2016; Cardoso et al., 2017). In this work, we showed Tap1a engendered inhibition through alike mechanism *via* interactions with the VSDII S3–S4 loop of Na_V_1.1. This mechanism was investigated using a K_V_2.1/Na_V_1.1 VSDII channel chimera, and our results showed Tap1a at nanomolar concentration interacted with the Na_V_1.1 VSDII S3–S4 loop which is in line with our previous findings with the wild-type Na_V_1.1 channel α subunit associated to the β1 subunit (Cardoso et al., 2021).

Remarkable work elucidated the structure-function and/or optimized the spider peptides CcoTx-1 (Shcherbatko et al., 2016), HwTx-IV (Minassian et al., 2013; Revell et al., 2013; Rahnama et al., 2017) and GpTx-1 (Murray et al., 2015; Murray et al., 2016), all belonging to the NaSpTx1 (Klint et al., 2012). Besides, CcoTx-1 and HwTx-IV were previously shown to preferably bind to VSDII S3-S4 loop of Na_V_ channels (Xiao et al., 2008; Xiao et al., 2010; Shcherbatko et al., 2016). The strong resemblance of mechanisms of action and structure-function properties of NaSpTx1 peptides encouraged studies of Tap1a, also in NaSpTx1 family, through select alanine scanning and guided rational design for the optimization of Na_V_-inhibitory properties.

In line with the NaSpTx1 family, Tap1a lost Na_V_ inhibitory activity by individual alanine substitutions of hydrophobic residues located at the centre of loop 1 and at the C-terminal, P and D in loop 2 and S and Q in loop 4. Contrary to HwTx-IV, the alanine substitution of a Q at the C-terminal of Tap1a enhanced its activity for Na_V_1.1 and nearly did not alter its activity for Na_V_1.7, indicating that Tap1a still maintain intrinsic properties associated to its structure-function relationships and unique potency and selectivity for Na_V_ subtypes. We suggested from our molecular docking studies that most of the Tap1a wild-type interactive surface is in the loop 4 and C-terminal, with fewer potential hydrophobic interactions in loop 1. In addition, the three-dimensional model of the Tap1a structure suggested the residues P12, D15 and Q28 were not located in the binding surface and this way are likely involved in the maintenance of Tap1a’s active structure. Experiments investigating the secondary and three-dimensional structures of Tap1a and derived analogues are essential to further elucidate the interactions between Tap1a and Na_V_ channels.

### Tap1a Binding Mode to Na_V_ Channels is Broadly Conserved in NaSpTx Peptides

The binding mode of NaSpTx peptides to Na_V_ channels has been studied in detail to unravel a conserved binding mechanism here named as “Lysin and Arginine electrostatic trap”. This mode uses strong electrostatic interactions driven by basic/positive charged lysin and arginine residues generally located in the loop 4 and C-terminal of a peptide that interacts with negatively charged residues such as glutamic acid and aspartic acid in S3–S4 loop of VSDII, and in which interactions are enhanced by vicinal hydrophobic interactions both within the channel and/or the cellular membrane. This has been shown in detail for NaSpTx1 peptides, including HwTx-IV (Wisedchaisri et al., 2021), GpTx-1 (Murray et al., 2016) and CcoTx-1 (Shcherbatko et al., 2016). For example, the binding of GpTx-1 to the hNav1.7 was studied by single substitutions and molecular docking, revealing the phenylalanine in loop 1 (F5) and the lysin in the C-terminal (K31) interacted with I767 in S2 and E811 of the S3–S4 loop of VSDII, respectively (Murray et al., 2016). In the present study, Tap1a wild-type showed a phenylalanine in loop 1 (F6) interacted with A766 in S2 and a lysin at the C-terminal (K31) interacted with E811 in the S3–S4 loop.

In Tap1a-OPT1, we hypothesized that these interactions were enhanced by the replacement of aspartic acid by lysin in position 33 which provided additional electrostatic interactions which are not present in the Tap1a wild-type. Similarly, in NaSpTx2 the peptide Pn3a showed interactions between K22 and K24 in loop 4 with the D816 and E818 in the S3–S4 segment predicted by molecular docking (Mueller et al., 2020). In NaSpTx-3, a similar mechanism was proposed for the peptide ProTx-II where R22 and K26 in loop 4 and C-terminal, respectively, interacted with E811 and D816 in S3–S4 loop of VSDII of hNa_V_1.7 (Xu et al., 2019). Overall, this conserved inhibition mechanism uses the insertion of positive charges often *via* lysin and arginine residues in the S3–S4 cleft of the VSDII to produce an electrostatic repulsion against S4 and the requirement for more positive potentials to induce the upward movement of S4 and the opening of the pore.

Although Cryo-EM and X-ray studies of Na_V_ channels have disclosed their three-dimensional structure in detail, these were mostly performed with channels at the open-state in which the voltage-sensor S4 is upwards and the channel pore open (Pan et al., 2018; Pan et al., 2019; Shen et al., 2019; Pan et al., 2021; Wisedchaisri et al., 2021), or did not describe key residues in the loop S3–S4 of VSDII which are essential for peptide binding (Shen et al., 2019). We performed docking experiments with Tap1a-WT and Tap1a-OPT1 on these available channel structures, and the models that produced closer interactions between VSDII and Tap1a used the structure determined for the complex hNa_V_1.7 VSDII-ProTx-II (Xu et al., 2019), and the hNa_V_1.4 channel structure determined in complex with the β1 auxiliary subunit (Pan et al., 2018). Our findings with these channel structures are consistent with the structure-function relationship findings for other NaSpTx peptides (Cardoso and Lewis, 2019; Shen et al., 2019; Xu et al., 2019; Mueller et al., 2020; Wisedchaisri et al., 2021).

### Tap1a was Optimized *via* SAR-Incorporation to Enhance Pharmacology *In Vitro* and Bioacivity *In Vivo*

Optimized Na_V_-targeting spider peptides display an increased affinity for the Na_V_ α subunit and the cell membrane, and hence improved potency. It is known the reduction in negative charge at the N-terminal, loop 1 or loop 3 of spider peptides can improve Na_V_ inhibition through an increase in affinity for the cell membrane (Henriques et al., 2016; Agwa et al., 2017). These occur in the NaSpTx1 and NaSpTx3 peptides (Cardoso and Lewis, 2019). In Tap1a-OPT1, the substitutions D1G and D2G at the N-terminal, D27H and Q28R at loop 4, and Q33K at the C-terminal likely increased its affinity for the cell membrane. These suggest that some residues could function by interacting with the cell membrane to initiate the binding process, and then by forming strong electrostatic interactions with the Na_V_ α subunit leading to the final binding fit.

The activity kinetics observed in our study implies the optimization of Na_V_ inhibition by Tap1a-OPT1 occurred *via* a significant slower off-rate, and this mechanism was conserved in both hNa_V_1.1 and hNa_V_1.7 subtypes. In a previous report, we demonstrated the enhancement of pharmacological Na_V_-inhibition by venom-derived bivalent ligand peptides produced *via* a similar slower off-rate mechanism that conferred improved potency at Na_V_1.4 (Peschel et al., 2020). Additional studies investigating lipids binding and ion channel interactions are essential to unravel if the slower off-rate is mediated *via* interactions with the Na_V_ α subunit and/or *via* interactions with the cell membrane.

An interesting observation from this study was the key role of lysine residues in the loop 4 and C-terminal to the binding to VSDII S3–S4 loop and inhibition of Na^+^ currents. Tap1a-WT displayed strong h-bonds formed by K26 and K31 and Na_V_1.7 VSDII, while the substitution K31A led a loss of activity for hNa_V_1.1 and hNa_V_1.7. In Tap1a-OPT1, h-bonds were formed by K31 and the new K33 in the C-terminal. Similarly, a study with the optimized peptide m3-HwTx-IV and the Na_V_1.7 performed *via* Cryo-EM revealed K27 in loop 4 and K32 at the C-terminal as the main drivers of this inhibitory mechanism (Wisedchaisri et al., 2021). Besides forming strong h-bonds, lysin residues can effectively interact with lipids in the cell membrane as demonstrated in studies with the peptide ProTx-II (Henriques et al., 2016). The substitution of lysine residues by arginine in ProTx-II led to a loss of affinity for lipids, and the substitution E17K led to a significant increase in affinity for lipids. These observations further support the hypothesis that key residues in ICK peptides can function initially as lipid binders to facilitate the initial encounter with the ion channel and later as ion channel binders to reach the final fit and nanomolar inhibition of the channel activity.

Because Tap1a-OPT2 lost at least 10-fold activity for Na_V_1.4 but only 2-fold activity for Na_V_1.7 compared to Tap1a-OPT1, we suggest that the phenylalanine residue in loop 1 of Tap1a-OPT1 is critical for an effective binding to Na_V_1.4. A study with GpTx-1 showed a similar trend for the analogue GpTx-F5A that showed 2.5-fold loss activity for Na_V_1.7 and 32.5-fold loss of activity for Na_V_1.4, and structural changes in K31 and Y32 (Lawrence et al., 2019). Interestingly, the analogue Tap1a-F7A completely lost its inhibition for Na_V_1.1 and Na_V_1.7 at up to 1 μM. Based on these observations, we suggest the phenylalanine in loop 1 is critical for the affinity of NaSpTx1 peptides for Na_V_1.4, while the affinity of NaSpTx1 peptides for Na_V_1.7 is less dependent on this residue. We must also consider the likeliness of structural changes Tap1a-F7A and Tap1a-OPT2 mutants, as observed for GpTx-1 F5A, which can allosterically affect the binding of vicinal residues to the ion channel. Further experiments *via* nuclear magnetic resonance to determine the three-dimensional structure of Tap1a and its derived analogues are essential to test this hypothesis.

The translation of the pharmacology optimized *in vitro* to enhanced bioactive effects *in vivo* has challenged studies with spider peptides targeting Na_V_ channels. For example, optimization of HwTx-IV towards lipid bilayers binding and Na_V_ inhibition did not result in the enhancement of bioactivity *in vivo* compared to HwTx-IV wild type (Agwa et al., 2020). In another study, the enhancement of Na_V_ inhibition observed *in vitro* by the addition of C-terminal amide to the spider peptide Df1a did not translate in enhanced *in vivo* effects (Cardoso et al., 2017). These studies suggest the C-terminal amidation could be associated to an enhancement in lipids affinity only and that this may not be sufficient to produce efficient inhibitory effects *in vivo* such as the interactions with the ion channel. In this present study, the rational optimization of Tap1a generating Tap1a-OPT1 produced a new therapeutic lead with remarkable and significant increase in bioactive effects *in vivo* compared to the Tap1a wild type. In addition, we designed Tap1a-OPT2 with selectivity away from Na_V_ off-targets and greater potential to provide lesser side effects in more complex animal models of diseases. Such results are very encouraging and can guide the rapid optimization of other peptide Na_V_ inhibitors to produce leads with useful improved *in vivo* therapeutic effects.

### Conclusions and Perspectives

Research for the development of novel drugs to tackle unmet needs in complex neurological diseases has progressed significantly in the last few years. Advancements in the understanding of disease mechanisms (Cardoso, 2020) and the structure function properties of key ion channel drug targets (Cardoso and Lewis, 2019; Mueller et al., 2020; Wisedchaisri et al., 2021) have provided key support for this progress. Although a significant number of ion channel modulators are available (Cardoso et al., 2018; Cardoso and Lewis, 2018), spider peptides have exceled in their therapeutic potential as proved by academic and industry research.

Spider peptides interact with the extracellular domains of voltage-gated ion channels and allosterically alter the channel conformation to facilitate (activator) or impede (inhibitor) the upward movement of voltage-sensor segments and the opening of the ion-selective pore. This modulatory mechanism is useful in the context of diseases with pathologies underlay by Na_V_-mediated dysfunctional neuronal signalling as observed for chronic visceral pain in IBS. For example, the spider peptide Hm1a, an allosteric activator of Na_V_1.1, produced chronic visceral pain-like symptoms in mice (Osteen et al., 2016), and the spider peptide Tap1a, an allosteric inhibitor of Na_V_ channels, reversed chronic visceral pain mediated by Na_V_ hyperfunction (Cardoso et al., 2021).

By applying the available structure-function relationship findings for spider peptides, we were able to rapidly produce an optimized spider peptide with enhanced *in vitro* pharmacology and enhanced *in vivo* bioactive effects. We observed this optimization produced a significant structure-function relationship improvement that likely generated a new and more efficient binding mode to the Na_V_ channel. The exact mechanisms through which this beneficial enhancement was achieved are still not fully clear, and we hypothesized it involved a combination of optimized interactions with the ion channel *via* the conserved “lysine and arginine electrostatic trap”, as well as with the lipidic cell membrane.

As perspectives from this work, further studies with chemically synthesized Tap1a analogues with C-terminal amide and non-natural amino acids to explore the chemical space available, the determination of their three-dimensional structure and binding sites on Na_V_ channels, along the evaluation of novel peptide leads in more relevant pre-clinical models of neurological diseases will expand this study to further support the research into novel therapeutics to treat complex neurological disorders.

## Materials and Methods

### Animals

For behavioral assessment we used adult male C57BL/6J mice aged 6–10 weeks. Mice were housed in groups of 4 per cage, under 12 h light-dark cycles and had standard rodent chow and water *ad libitum*. Ethical approval for *in vivo* experiments was obtained from the University of Queensland animal ethics committee. All experiments were conducted in accordance with the International Association for the Study of Pain Guidelines for the Use of Animals in Research, and the Australian Code of Practice for the Care and Use of Animals for Scientific Purposes, 8th edition (2013, updated 2021).

### Cell Culture

HEK293 cells were cultured in Dulbecco’s MEM supplemented with 10% FBS, 100 U.ml^−1^ penicillin and 100 μg.ml^−1^ streptomycin. HEK293 cells stably expressing recombinant hNa_V_ subtypes and the β1 auxiliary subunit (SB Drug Discovery, Glasgow, United Kingdom) were cultured in Minimal Essential medium (MEM) (Sigma-Aldrich, MO, United States) supplemented with 10% FBS, 100 U.ml^−1^ penicillin, 100 μg.ml^−1^ streptomycin, 2 mM L-glutamine, and variable concentrations of blasticidin, geneticin and zeocin according to manufacturer’s instructions. All cells were maintained at 37°C in a humidified 5% CO_2_ incubator, and subcultured every 3–4 days in a ratio of 1:5 using 0.25% trypsin/EDTA.

### Patch-Clamp Electrophysiology of Na_V_1.1/K_V_2.1 Chimeric Channel

The plasmid constructs containing the voltage sensor extracellular domains II of the Na_V_1.1 channel inserted into the K_V_2.1 channel was kindly provided by Prof Frank Bosman from the Department of Basic and Applied Medical Sciences, Ghent University, Belgium (Osteen et al., 2016). The Na_V_1.1/K_V_2.1 chimera was subcloned into the mammalian vector pCDNA3.1 and new constructs confirmed by DNA sequencing. Plasmid constructs were used to transfect HEK293 cells using the FuGENE reagent as previously described (Hasan et al., 2020). Briefly, HEK293 cells were transfected with 18 μg DNA using a ration of DNA:FuGENE at 1:3 and transfection media replaced with fresh media after 16 h incubation at 37°C 5% CO_2_. After 48 h transfection, cells were used in the electrophysiology assays in the automated whole-cell patch clamp system QPatch 16X (Sophion Bioscience).

The extracellular solution comprised 140 NaCl, 5 KCl, 10 CaCl_2_, 2 MgCl_2_, 10 glucose and 10 HEPES at pH 7.4 and 320 mOsm. The intracellular solution comprised (in mM) 150 KCl, 1 MgCl_2_, 4 NaCl, 0.5 EGTA and 10 HEPES at pH 7.4 and 320 mOsm. Cells were maintained at a holding potential –90 mV and K^+^ currents elicited by +20 mV pulse for 500 ms followed by –40 mV pulse for additional 500 ms. Tap1a-WT was added at increasing concentrations from 1.4 nM to 1 μM, while nifedipine was added at a single concentration of 100 μM. Electrophysiological data were analyzed using QPatch Assay Software v5.6 (Sophion Bioscience).

### Patch-Clamp Electrophysiology of Na_V_ Channels

Na_V_ channels currents were recorded from HEK293 cells expressing human Na_V_ subtypes co-expressed with the β1 auxiliary subunit (SB Drug Discovery) using the automated whole-cell patch clamp system QPatch 16X (Sophion Bioscience A/S, Ballerup, Denmark). The extracellular solution comprised (in mM) 1 CaCl_2_, 1 MgCl_2_, 5 HEPES, 3 KCl, 140 NaCl, 0.1 CdCl_2_, 20 TE A-Cl, at pH 7.3 and 320 mOsm. The intracellular solution comprised (in mM) 140 CsF, 1/5 EGTA/CsOH, 10 HEPES, 10 NaCl at pH 7.3 and 320 mOsm.

Cells were maintained at a holding potential –80 mV and Na^+^ currents elicited by 20 ms voltage steps to 0 mV from a –120 mV conditioning pulse applied for 200 ms. Voltage-activation relationships were obtained by measuring steady-state Na^+^ currents elicited by step depolarizations from –110 to +80 mV using 5-mV increments. The peak conductance (*G*_Na_) was calculated from *G* = *I*/(*V*–*V*_rev_), where *I*, *V* and *V*_rev_ represent the current value, membrane potential, and reversal potential, respectively. The voltage of steady-state fast inactivation was estimated using a double-pulse protocol with currents elicited by a 20 ms depolarizing potential of 0 mV from a 500 ms pre-pulse to potentials ranging from –130 to –10 mV using 10-mV increments.

To obtain concentration–response curves, cells at holding potential were incubated for 5 min with increasing concentrations of Tap1a-OPT1 and Tap1a-OPT2. The voltage-dependence of activation and inactivation were determined either in the absence or following 5-min exposure to Tap1a-OPT1 at the respective IC_50_ concentration. For on-rate experiments, Na^+^ currents were measured at 15 s intervals over 15 min immediately following the addition of peptide at a concentration equivalent to 10 times their IC_50_ for the Na_V_ subtype being analysed. For off-rate measurements, cells were incubated with peptide for 10 min at a concentration equivalent to 10 times their IC_50_ for the Na_V_ subtype being analysed, and Na^+^ currents were assessed at 10-s intervals during saline washes. The *k*_on_, *k*_off_ and *K*_d_ values were calculated using *K*_d_ = *k*_off_/*k*_on_ (nM), where *k*_off_ = 1/τ_off_ (s^−1^) and *k*_on_=[(1/τ_on_)−K_off_]/(Maertens et al., 2006) (nM^−1^S^−1^). Data were analysed using Assay software (Sophion Biosciences) and Na^+^ currents (*I*_Na_) plotted as *I*/*I*max.

### Alanine Scanning of Tap1a

Alanine substitutions in the primary sequence of Tap1a were made based in structure-activity relationship (SAR) studies of other spider peptides belonging to NaSpTx1 (Cardoso and Lewis, 2019). The alanine mutants were produced using the QuikChange Lightning site-directed mutagenesis kit and the Tap1a-pLICC plasmid construct as directed by the manufacturer (Agilent, CA, United States).

Briefly, primers containing the substitutions M6A, F7A, P12A, D15A, S24A, D27A, Q28A, W29A, K31A, Y32A, Q33A, L34A and W35A were designed and used in polymerase chain reactions (PCR) to generate alanine mutants. After digestion with the restriction enzyme DpnI to eliminate the Tap1a wild-type construct from the reaction, the newly synthesized mutants were transformed into *E. coli* strain DH5-α and plated in LB agar containing 100 μg.ml^−1^ ampicillin, 80 μg.ml^−1^ X-gal and 20 mM IPTG. White colonies were collected, and plasmid DNA extracted using QIAprep Spin Miniprep kit (QIAGEN, Hilden, DEU) as directed by the manufacturer. The new Tap1a-alanine mutants constructs were confirmed by DNA sequencing.

### Rational Design of Optimized Tap1a

The rational design of Tap1a used the available information on the structure-activity relationship (SAR) studies of other spider peptides belonging to NaSpTx1 (Cardoso and Lewis, 2019) (Figure 3A). The following substitutions to the primary sequence of Tap1a were made to create Tap1a-OPT1: D1G, D2G, M6I, N13E, P19Y, K22V, R25K, D27D, Q28R, Y32W and Q33K. Synthetic gene encoding Tap1a-OPT1, with codons optimized for expression in *E. coli*, was produced and subcloned into the pLICC vector by GeneArt (Life Technologies, CA, United States). The Tap1a-OPT2 expression plasmid was generated by the replacement F7A in Tap1a-OPT1 using the QuikChange Lightning site-directed mutagenesis kit as described above. The new Tap1a-OPT2 construct was confirmed by DNA sequencing.

### Production of Recombinant Peptides

The peptide mutants were produced using the QuikChange Lightning site-directed mutagenesis kit and the Tap1a-pLICC plasmid construct as directed by the manufacturer (Agilent, CA, United States) with codons optimized for expression in *Escherichia coli* and subcloned into the pLICC vector by GeneArt (Life Technologies, CA, United States). This expression vector contains a MalE signal sequence for periplasmic export, a His_6_ tag fused to the maltose binding protein (MBP), and a tobacco etch virus (TEV) protease recognition site preceding the peptide gene. Peptide expression was performed as described previously (Cardoso et al., 2015).

Briefly, plasmids were transformed into the protease-deficient *E. coli* strain BL21 (DE3), then cells were cultured at 37°C in Luria-Bertani medium. Peptide expression was induced at OD_600_ = 1 with 0.5 mM isopropyl β-D-1-thiogalactopyranoside (IPTG), then cells were harvested after 16 h at 16°C and pelleted by centrifugation for 20 min at 10,000 rpm. Cells were lysed using FastBreak lysis buffer (Promega, WI, United States) containing 0.2 mg.ml^−1^ lysozyme and 10 U.ml^−1^ DNAse.

The fusion protein was captured by passing the cell lysate through a NI-NTA resin (Sigma-Aldrich, Aldrich, MO, United States), then the His_6_-MBP-toxin fusion protein was eluted with 500 mM imidazole in TN buffer (25 mM Tris, 150 mM NaCl, pH 8). After desalting, the purified fusion protein was cleaved with TEV protease, then the peptide was purified using RP-HPLC performed using an Ultimate 3000 LC system (Dionex, Sunnyvale, CA, United States) with a C18 column (Vydac 4.6 mm × 250 mm, 5 μM). The peptide was eluted using a gradient of 10–50% solvent B in Solvent A, using a flow rate of 0.7 ml.min^−1^. Peaks were collected at 0.7 ml per well, then fractions lyophilized and stored at –20°C.

### OD1-Induced Spontaneous Pain

The Na_V_1.7 activator OD1 (300 nM) was diluted in saline/0.1% BSA and administered by intraplantar injection into the left hind paw of adult (6–10 weeks) male C57BL/6 mice under light isoflurane (4%) anesthesia as previously described (Cardoso et al., 2015; Deuis et al., 2016). Tap1a WT (1 μM) or Tap1a OPT (1 μM) were co-administered by intraplantar injection with OD1. Immediately after injection mice were placed in polyvinyl boxes (10 × 10 × 10 cm), and spontaneous pain behaviors, which included flinching, licking, and shaking of the injected hind paw, were recorded by video and later counted by a blinded observer unaware of the treatment each individual mouse received.

### Molecular Docking

The molecular docking of Tap1a-WT and Tap1a-OPT1 over the hNa_V_1.7 VSDII was performed using HADDOCK2.4 (van Zundert et al., 2016) and visualized and analyzed using PyMol (DeLano, 2002). The Tap1a-WT, Tap1a-OPT1 and Tap1a-OPT2 molecular models were produced using as template the NMR structure of the tarantula toxin μ-TRTX-Pre1a-W7A (PDB 512P, unpublished results, 71.4% identity with Tap1a-WT), and models generated in SWISS-MODEL (Arnold et al., 2006).

For the molecular docking experiments over hNa_V_1.7, the cryo-EM structure of the chimera hNa_V_1.7VSDII-Na_V_Ab on its deactivated state and S4 downwards was used (PDB 6N4R) (Xu et al., 2019). Residues involved in the bioactivity of Tap1a-WT for Na_V_1.7 channels determined using an alanine scanning (M6, F7, P12, D15, S24, D27, Q28, K31, Y32, Q33, L34 and W35) or rational optimization (G1, G2, I6, E13, Y19, V22, K25, H27, R28, W32 and K33) were used as restrains. Additional restrains were applied at F812, D815 and E817 located in the Na_V_1.7 VSDII S3-S4 loop which were previously described to participate in the interactions with spider peptides (Xiao et al., 2008; Xiao et al., 2010).

For the molecular docking experiments over hNa_V_1.4, the cryo-EM structure of the human Nav1.4 was used (PDB 6AGF) (Pan et al., 2018). Modified residues in the sequences of Tap1a-OPT1 (G1, G2, I6, E13, Y19, V22, K25, H27, R28, W32 and K33) and Tap1a-OPT2 (OPT1 plus A7) were used as restrains. Additional restrains were applied at N661 and Q663 located in the Na_V_1.4 voltage-sensor domain II S3–S4 loop and previously described to participate in the interactions with spider peptides (Xiao et al., 2008; Xiao et al., 2010).

The HADDOCK cluster result displaying the most negative HADDOCK Z-score was selected for further analysis. H-bond interaction distances in Å were calculated automatically in Pymol, while hydrophobic interaction distances were calculated manually in Pymol using as criteria the closest distance between the carbons located in the side chains. The electrostatic surface potential of Tap1a-WT, Tap1a-OPT1 and hNav1.7 DII were calculated using the linearized Poisson-Boltzmann equation through the APBS Electrostatics plugin for PyMol (Dolinsky et al., 2004).

### Data Analysis

For the *in vitro* electrophysiological recordings, curve fitting was performed using GraphPad Prism Version 8 (GraphPad Software, San Diego, CA, United States) using nonlinear regression with log-inhibitor versus normalized response and variable Hill slope for dose-responses and IC_50_ determination, and exponential one-phase association and dissociation for on and off-rate analysis, respectively. Potencies expressed in IC_50_ values were compared amongst Tap1a-WT, Tap1a-OPT1 and Tap1a-OPT2 using multiple *t*-student test with Welch correction. The kinetics of interaction and dissociation were compared amongst Tap1a-WT, Tap1a-OPT1 and Tap1a-OPT2 using Mann-Whitney test. Data are mean ± SEM, *n* = number of individual cells recorded. For the *in vivo* experiments, statistical significance was determined by one-way ANOVA with Dunnett’s post-test and data was presented as mean ± SEM, *n* = 4 per mice per group.

## Data Availability

The original contributions presented in the study are included in the article/Supplementary Material, further inquiries can be directed to the corresponding author.
