# Supplementary material for: Engineering of a Spider Peptide via Conserved Structure-Function Traits Optimizes Sodium Channel Inhibition In Vitro and Anti-Nociception In Vivo
Source: Front Mol Biosci. 2021 Sep 21;8:742457. doi: 10.3389/fmolb.2021.742457 (PMC8490825; doi:10.3389/fmolb.2021.742457)

Supplementary Material

Supplementary Table 1. Predicted and observed molecular weight of recombinant Tap1a alanine mutants designed and produced in this work. Reported masses are for the monoisotopic molecular weight determined for the purified recombinants with an additional N-terminal glycine.

Tap1a alanine mutant	Predicted M.W. (Da)	Observed M.W. (Da)
M6A	4176.7	4176.7
F7A	4160.7	4160.8
P12A	4120.7	4210.6
D15A	4192.7	4192.7
S24A	4220.7	4220.6
D27A	4192.7	4192.7
Q28A	4179.7	4179.7
K31A	4179.7	4179.7
Y32A	4144.7	4144.7
Q33A	4179.7	4179.7
L34A	4194.7	4194.8
W35A	4121.7	4121.7

Supplementary Table 2. Potential interactions of Tap1a-WT, Tap1a-OPT1 and OPT2 with Nav1.7 or Nav1.4 determined by molecular docking. Hydrophobic interactions distances were calculated between the closest carbons of their side chains and had cut-off 5.0 Å, while hydrogen bonds distances had cut-off 3 Å. Hydrogen bonds and hydrophobic interactions distances were predicted using PyMol. The results are from docking clusters displaying the most negative HADDOCK Z-core.

Nav1.7

Alanine scan restrains

Tap1a-WT residue	Nav1.7 DII residue	Hydrogen bond (Å)	Tap1a-WT residue	Nav1.7 DII residue	Hydrophobic interactions (Å)
R25	E817	2.9	F7	A766	3.9
K26	E817	1.5	Y32	A766	3.4
D27	R823	1.6 / 1.6	Y32	L770	3.7
Q28	E817	2.1	W35	F812	3.6
K31	E810	1.9 / 2.2	W35	L813	3.5
K31	L813	1.8			
Y32	A766	2.0			
Q33	L813	2.0			
Q33	L811	2.9			

Optimization restrains

Tap1a-OPT1 residue	Nav1.7 DII residue	Hydrogen bond (Å)	Tap1a-OPT1 residue	Nav1.7 DII residue	Hydrophobic interactions (Å)
S24	D815	2.7	V22	F812	3.2
S24	A814	1.8	V22	L813	4.0
W29	D815	2.9	W29	V816	4.1
K31	D815	1.8	W35	I767	4.5
K31	E817	2.0	W35	L770	3.8
W32	R823	2.1			
K33	E810	2.4			
K33	L811	1.6			
K33	F812	2.1			
K33	L813	1.7			

Tap1a-WT residue	Nav1.7 DII residue	Hydrogen bond (Å)	Tap1a-WT residue	Nav1.7 DII residue	Hydrophobic interactions (Å)
K22	L811	1.7	W29	V816	5.2
K22	L813	1.8	L34	A766	4.1
S24	A814	1.8 / 2.4	W35	L770	4.2
K31	E817	1.6	W35	I767	4.2
K31	D815	1.9 / 2.1 / 2.5			

Na$_V$1.4

Optimization restrains

Tap1a-OPT1 residue	Nav1.4 DII residue	Hydrogen bond (Å)	Tap1a-OPT1 residue	Nav1.4 DII residue	Hydrophobic interactions (Å)
P12	N1204	1.8	W32	V662	4.7
E13	S1207	2.7	L34	V662	2.7/4.1
K25	N1205	2.6	L34	L665	4.2
K25	E1260	1.6/2.1			
K31	D661	2.1			
K33	Q663	1.8			
K33	E1258	1.6			

Tap1a-OPT2 residue	Nav1.4 DII residue	Hydrogen bond (Å)	Tap1a-OPT2 residue	Nav1.4 DII residue	Hydrophobic interactions (Å)
S24	N661	1.9	W29	A660	2.1/3.4
K25	E1258	1.6			
K26	S666	2.5			
K26	V662	2.0			
K26	L665	1.9			
R28	D607	1.6/1.6			
W29	A660	2.1			
K31	L659	1.8			

Alanine scan restrains

Tap1a-WT residue	Nav1.4 DII residue	Hydrogen bond (Å)	Tap1a-WT residue	Nav1.4 DII residue	Hydrophobic interactions (Å)
K22	E656	1.7 / 1.8	Y32	A660	4.1
S24	G658	2.3			
R25	L657	2.0			
K31	N661	1.6			
Q33	V662	1.9			

Supplementary Figure 1. Chimera hNa$_V$1.1/rK$_V$2.1 containing the paddles S3-S4 from Na$_V$1.1 DII. Potassium currents elicited by depolarization to +20 mV are shown before (black trace) and after (red trace) addition of nifedipine 100 μM. Endogenous potassium channels in non-transfected HEK293 cells elicited by depolarization to +20 mV are shown in green. Data are representatives from *n* = 3 independent experiments for each condition assayed, one whole cell was considered per independent experiment.

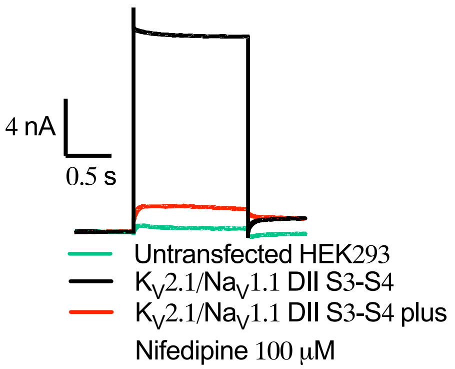

Supplementary Figure 2. Representatives of the recombinant expression in *E. coli* and purification of individual selected Tap1a alanine mutants with substitutions in the loops 1, 3, 4 or C-terminal. Left: Chromatograms of the purified Tap1a alanine mutant recombinant peptides analyzed by RP-HPLC in C18 column and using 20% to 50% B gradient. Right: Mass spectrometry results of the Tap1a alanine peptides after purification by RP-HPLC. Masses are represented as $[M/Z]^{+z}$.

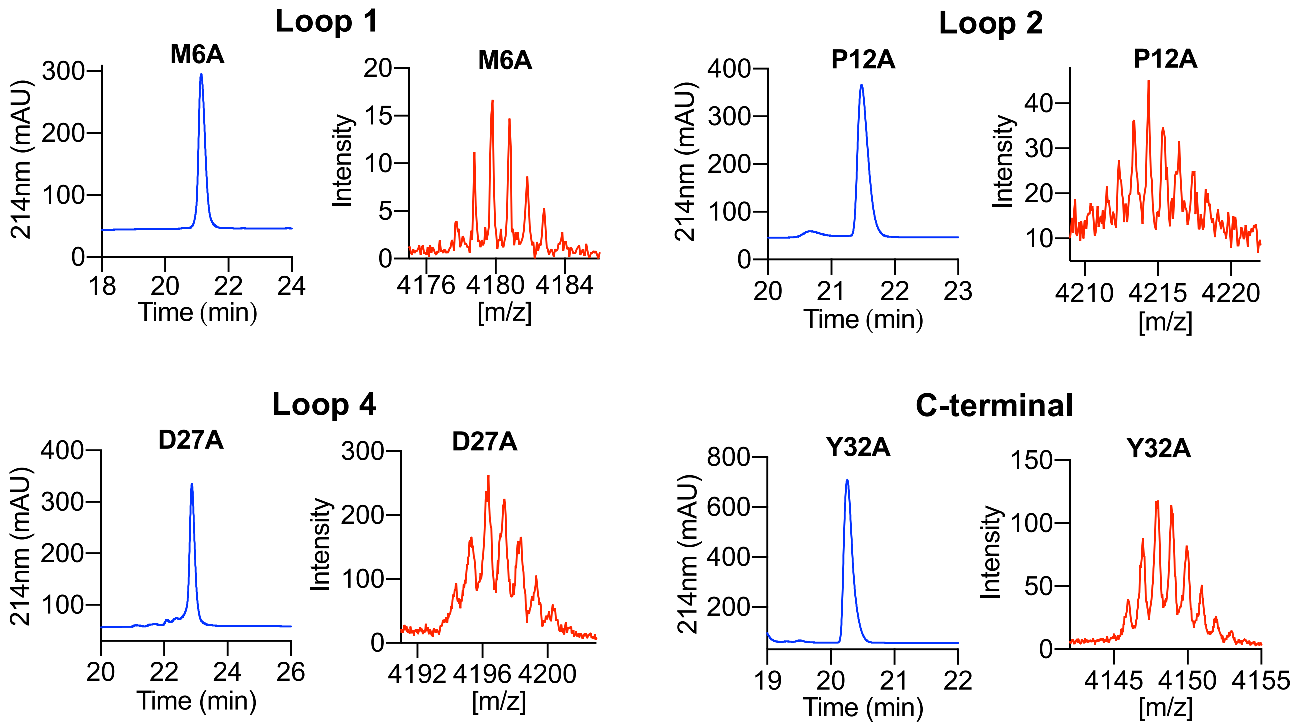

Supplementary Figure 3. Molecular docking results obtained from Haddock experiments showing the score achieved by each selected cluster discussed in this work.

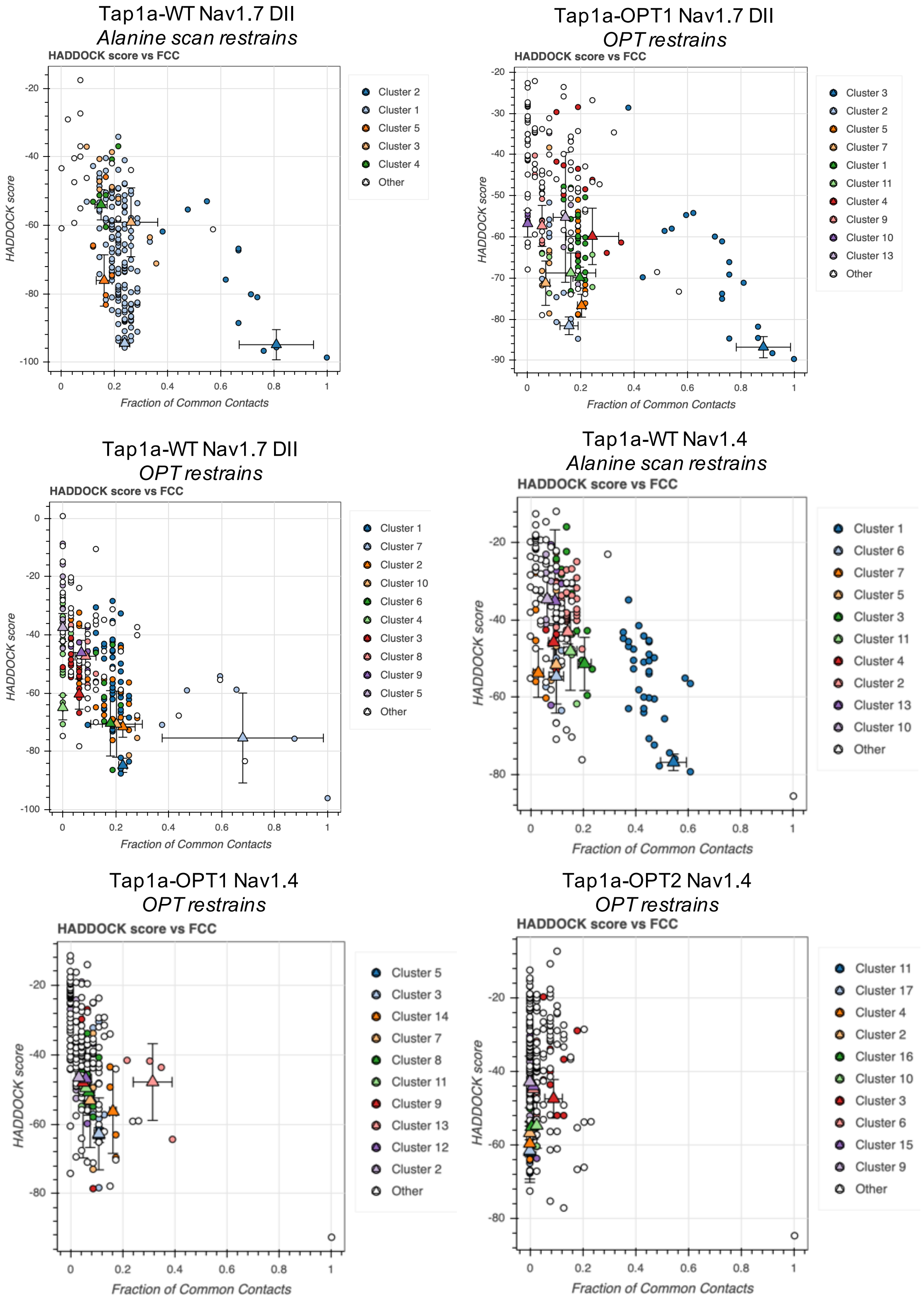

Supplement: Supplementary file 1 [file DataSheet1.PDF]
